# Prospective Validation of ELF Test in Comparison with Fibroscan and FibroTest to Predict Liver Fibrosis in Asian Subjects with Chronic Hepatitis B

**DOI:** 10.1371/journal.pone.0041964

**Published:** 2012-07-27

**Authors:** Beom Kyung Kim, Hyon Suk Kim, Jun Yong Park, Do Young Kim, Sang Hoon Ahn, Chae Yoon Chon, Young Nyun Park, Kwang-Hyub Han, Seung Up Kim

**Affiliations:** 1 Department of Internal Medicine, Yonsei University College of Medicine, Seoul, Korea; 2 Institute of Gastroenterology, Yonsei University College of Medicine, Seoul, Korea; 3 Department of Laboratory Medicine, Yonsei University College of Medicine, Seoul, Korea; 4 Department of Pathology, Yonsei University College of Medicine, Seoul, Korea; 5 Liver Cirrhosis Clinical Research Center, Yonsei University College of Medicine, Seoul, Korea; 6 Brain Korea 21 Project for Medical Science, Yonsei University College of Medicine, Seoul, Korea; Lund University Hospital, Sweden

## Abstract

**Background and Aims:**

Liver stiffness measurement (LSM) and FibroTest (FT) are frequently used as non-invasive alternatives for fibrosis staging to liver biopsy. However, to date, diagnostic performances of Enhanced Liver Fibrosis (ELF) test, which consists of hyaluronic acid, aminoterminal propeptide of procollagen type-III, and tissue inhibitor of matrix metalloproteinases-1, have not been compared to those of LSM and FT in Asian chronic hepatitis B (CHB) patients.

**Methods:**

Between June 2010 and November 2011, we prospectively enrolled 170 CHB patients who underwent liver biopsies along with LSM, FT, and ELF. The Batts system was used to assess fibrosis stages.

**Results:**

Areas under receiver operating characteristic curves (AUROCs) to predict significant fibrosis (F≥2), advanced fibrosis (F≥3), and cirrhosis (F = 4) were 0.901, 0.860, and 0.862 for ELF, respectively; 0.937, 0.956, and 0.963 for LSM; and 0.896, 0.921, and 0.881 for FT. AUROCs to predict F≥2 were similar between each other, whereas LSM and FT had better AUROCs than ELF for predicting F≥3 (both *p*<0.05), and LSM predicted F4 more accurately than ELF (*p*<0.05). Optimized cutoffs of ELF to maximize sum of sensitivity and specificity were 8.5, 9.4, and 10.1 for F≥2, F≥3, and F = 4, respectively. Using suggested ELF, LSM and FT cutoffs to diagnose F1, F2, F3, and F4, 91 (53.5%), 117 (68.8%), and 110 (64.7%) patients, respectively, were correctly classified according to histological results.

**Conclusions:**

ELF demonstrated considerable diagnostic value in fibrosis staging in Asian CHB patients, especially in predicting F≥2. However, LSM consistently provided better performance for predicting F≥3 and F4.

## Introduction

Accurate assessment of the severity of liver fibrosis in patients with chronic hepatitis B (CHB) is necessary for not only prediction of the long-term clinical course, but also determination of whether and when to begin antiviral therapy. The most recent guidelines on the management of CHB proposed that the presence of significant fibrosis with detectable serum hepatitis B virus (HBV) DNA indicates antiviral therapy, since viral suppression can reduce liver-related complications in patients with CHB who have significant fibrosis to cirrhosis [1], [2]. Conversely, in patients without significant fibrosis and low levels of circulating virus, it is more appropriate to monitor rather than initiate expensive and potentially long-lasting antiviral therapy [1]. Furthermore, since patients with cirrhosis should be entered into the active surveillance program for early detection of hepatocellular carcinoma (HCC) and other complications associated with hepatic decompensation, including gastroesophageal varices, assessment of fibrosis in patients with CHB has become an important clinical issue for physicians [3].

To-date, liver biopsy has been the gold standard to assess liver fibrosis. It is often limited, however, by its invasiveness, cost, risk of complications, poor acceptance by patients, lack of availability of expert practitioners, and intra/inter-observer variability [4]. These drawbacks have also made sequential liver biopsies unfeasible, especially when repeated examinations are required to monitor the response to antiviral or anti-fibrosis treatment. Consequently, noninvasive approaches combining several biochemical parameters have been introduced, including aspartate aminotransferase (AST)-to-platelet ratio index (APRI) [5], AST-alanine aminotransferase (ALT) ratio [6], Forns test [7], and FibroTest (FT; BioPredictive, Paris, France) [8]. Among them, FT is a commercially available, popular, and non-invasive surrogate, which had been substantially validated in Caucasian patients with chronic hepatitis C (CHC), and thereafter in Asian subjects with CHB [9], [10], [11], [12], [13]. Meanwhile, liver stiffness measurement (LSM) using transient elastography (TE; FibroScan®; Echosens, Paris, France), which calculates liver elasticity using a low frequency elastic wave transmitted through the liver, has been introduced recently and proven useful for non-invasive assessment of liver fibrosis among subjects with chronic liver diseases (CLDs) due to various etiologies [14], [15], [16].

In 2004, the Original European Liver Fibrosis panel of serum markers of liver fibrosis incorporates hyaluronic acid (HA), tissue inhibitor of matrix metalloproteinases-1 (TIMP-1), and aminoterminal propeptide of procollagen type III (P3NP), all of which are involved in the synthesis and degradation of the extracellular matrix. It showed good diagnostic accuracy for the detection of moderate and severe fibrosis in a cohort of patients with mixed etiology CLDs, mainly due to hepatitis C virus (HCV) infection (49%) [17]. Thereafter, it was simplified by removing age while maintaining diagnostic accuracy, to establish the Enhanced Liver Fibrosis Test (ELF, Siemens Diagnostics, NY, USA) [18]. This can accurately predict significant liver fibrosis in independent populations [18], [19], [20], [21]. However, in contrast to other non-invasive surrogate markers available in the current clinical practice, few studies have investigated the diagnostic performance of ELF test in patients with CHB [22]. In particular, no previous study has focused on Asian patients with CHB.

Here, the present study prospectively compared the diagnostic value of ELF test in Asian populations with CHB with that of LSM and FT, two well-known non-invasive alternatives to liver biopsy, and defined optimized thresholds for prediction of liver fibrosis.

## Materials and Methods

### Patients' eligibility

From the database of the liver cirrhosis clinical research center at Severance Hospital, Yonsei University College of Medicine, Seoul, Korea, consecutive patients with CHB who underwent liver biopsy along with ELF test, LSM and FT on the same day between June 2010 and November 2011, were selected for this study. Liver biopsy was performed to assess the severity of fibrosis and inflammation prior to antiviral therapy.

**Figure 1:**
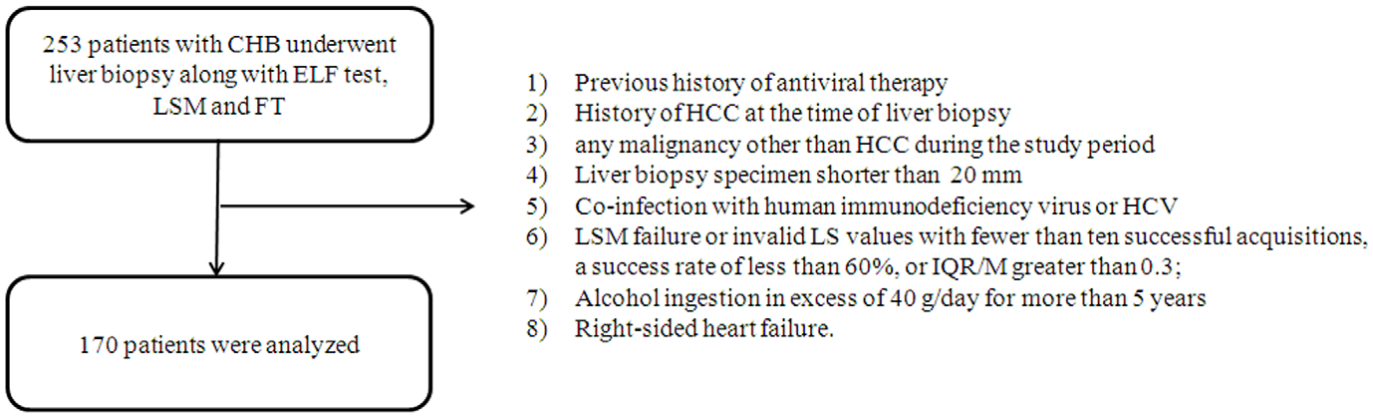
Flow chart describing the selection of the study population. Based on the exclusion criteria, 170 subjects were finally recruited for analyses.

The exclusion criteria were: previous history of antiviral therapy; the presence of HCC or history of it at the time of liver biopsy; any malignancy other than HCC during the study period; liver biopsy specimen smaller than 20 mm; co-infection with human immunodeficiency virus or HCV; LSM failure or invalid liver stiffness (LS) values with fewer than ten successful acquisitions, a success rate of less than 60%, or interquartile range (IQR)/median value ratio (IQR/M) greater than 0.3; alcohol ingestion in excess of 40 g/day for more than 5 years; or right-sided heart failure (Figure 1).

This study was performed in accordance with the ethical guidelines of the 1975 Declaration of Helsinki. Written informed consent was obtained from each participant or responsible family member after possible complications of the diagnostic procedures had been explained fully. This study was approved by the institutional review board of Severance Hospital.

### Liver biopsy examination

Percutaneous liver biopsy was performed using a 16-g disposable needle immediately following LSM. The liver biopsy specimens were fixed in formalin and embedded in paraffin. Then, sections 4 µm thick were stained with hematoxylin and eosin (H&E) and Masson's trichrome. All liver tissue samples were evaluated by an experienced hepatopathologist who was blinded to the patients' clinical histories. Specimens that were smaller than 20 mm or considered by the pathologists to be unsuitable for fibrosis assessment were excluded from the analysis. Liver histology was evaluated semi-quantitatively according to the Batts and Ludwig scoring system [23]. Fibrosis was staged on a 0–4 scale: F0, no fibrosis; F1, portal fibrosis; F2, periportal fibrosis; F3, septal fibrosis; and F4, cirrhosis. Significant fibrosis was defined as F2 or more and advanced fibrosis as F3 or more.

### ELF test

On the same day as LSM and liver biopsy, fasting blood samples were obtained and the serum was stored at −80°C. PIIINP, HA and TIMP-1 were measured using an ADVIA Centaur XP automated immunoanalyzer (Siemens Healthcare Diagnostics, Tarrytown, NY, USA). The ELF score was calculated using the algorithm recommended in the CE-marked assay [ELF = 2.278+0.851 ln(HA)+0.751 ln(PIIINP)+0.394 ln(TIMP-1)].

### FT score

On the same day as LSM and liver biopsy, the FT score parameters, including α2-macroglobulin, apolipoprotein A1, haptoglobin, γ-GGT, and total bilirubin, were assessed. The FT score was computed on the BioPredictive website (www.biopredictive.com) as follows: f = 4.467×log[α2-macroglobulin (g/L)]−1.357×log[haptoglobin (g/L)]+1.017×log[γ-GGT (IU/L)]+0.0281×[age (in years)]+1.737×log[bilirubin (µmol/L)]−1.184×[apolipoprotein A1 (g/L)]+0.301×sex (female = 0, male = 1)−5.540.

### LSM

LSM was performed by one well-trained technician blinded to patients' clinical and laboratory data on the same day as liver biopsy and the laboratory studies, including ELF test and FT. Details of the technique and examination procedure have been published previously [24], [25]. The results were expressed in kilopascals (kPa). IQR was defined as an index of intrinsic variability of LS values corresponding to the interval of the LS results containing 50% of the valid measurements between the 25^th^ and 75^th^ percentiles. The median value was considered representative of the elastic modulus of the liver. Only procedures with at least 10 valid measurements, a success rate of at least 60%, and an IQR/M <30% were considered reliable.

### Statistical analyses

The major goals of this study were to prospectively validate the diagnostic performance of ELF test to detect histologically confirmed significant fibrosis, advanced fibrosis, and cirrhosis and compared with that of LSM and FT, and to suggest optimal cutoff values of ELF test for Asian patients with CHB. To assess the diagnostic performance of each non-invasive index, receiver operating characteristic (ROC) curves were constructed and the areas under the ROC curves (AUROCs) were calculated. Then, to evaluate the usefulness of the non-invasive method, the sensitivity, specificity, positive predictive value (PPV), and negative predictive value (NPV) were determined from the ROC curves. The Hanley and McNeil test was used to compare the AUROC between two non-invasive models [26]. The most discriminant cutoff values were determined from the ROC curves to maximize the sum of sensitivity and specificity [27].

Statistical analyses were performed using SAS software, version 9.1.3 (SAS, Cary, NC). In all analyses, *p*<0.05 was considered statistically significant.

## Results

### Patients' baseline characteristics

A total of 253 consecutive patients were screened for possible inclusion in the study. Based on the exclusion criteria, a total of 170 patients (mean age 45.3 years, 102 male) were included (**Figure 1**).

The patients' characteristics are summarized in **Table 1**. The mean AST level was 45.9 IU/L, while the mean ALT level was 62.9 IU/L. The mean value of the ELF test was 9.56±1.69, while those of the LSM and FT were 12.24±7.76 kPa and 0.55±0.30, respectively. The mean length and the median number of fragments of liver biopsy samples were 21.3 mm and 2, respectively. The fibrosis stages were F0 in 10 (5.9%) patients, F1 in 39 (22.9%), F2 in 36 (21.2%), F3 in 38 (22.4%), and F4 in 47 (27.6%). All patients had adequate liver function.

**Table 1:** Baseline characteristics (n = 170).

Characteristics	Value
**Demographic data**	
Age (years)	45.3±15.1
Male gender, no. (%)	102 (60.0)
Body mass index (kg/m^2^)	23.4±2.8
**Laboratory data**	
Serum albumin (g/dL)	4.2±0.51
Total bilirubin (mg/dL)	1.26±0.90
Aspartate aminotransferase (IU/L)	45.9±21.3
Alanine aminotransferase (IU/L)	62.9±26.1
Prothrombin time (%)	90.2±13.9
Platelet count (10^9^/L)	183.9±73.3
**Biopsy length (mm)**	21.3±0.7
**Enhanced liver fibrosis test**	9.56±1.69
**Liver stiffness (kPa)**	12.24±7.76
**FibroTest**	0.55±0.30
**Fibrosis stage, no. (%)**	
F0	10 (5.9%)
F1	39 (22.9%)
F2	36 (21.2%)
F3	38 (22.4%)
F4	47 (27.6%)

Values were expressed as mean ± standard deviation, unless indicated otherwise.

### Diagnostic performances of the ELF test in comparison with LSM and FT

**Figure 2:**
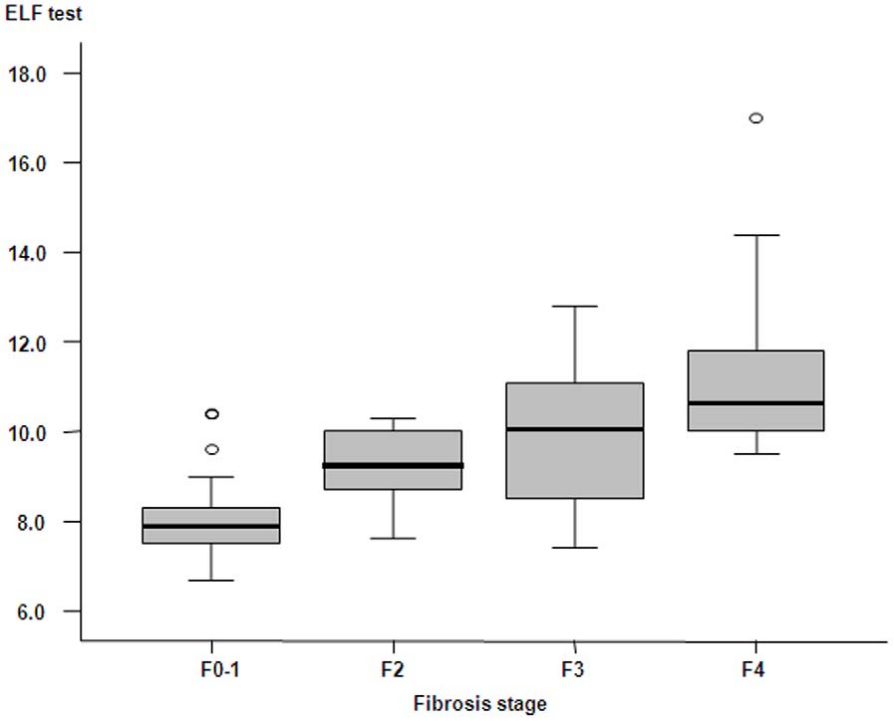
Box plots of ELF test according to fibrosis stage. Boxes and horizontal lines within boxes represent interquartile ranges (IQRs) and median values, respectively. The upper and lower whiskers indicate 75^th^ percentile plus 1.5 IQR and 25^th^ percentile minus 1.5 IQR, respectively. o, mild outlier: a value more than 75^th^ percentile plus 1.5 IQR, but less than 75^th^ percentile plus 3.0 IQR.

As shown in **Figure 2**, the overall mean values of ELF test increased parallel to the stage of fibrosis (8.01±0.84 in F0-1, 9.24±0.77 in F2, 10.03±1.54 in F3, and 11.10±1.49 in F4, all *p*<0.05 between adjacent fibrosis stages). The Spearman correlation coefficient of ELF test with histological fibrosis stages was 0.724 (*p*<0.001). Similarly, the mean value of the LSM and FT also significantly increased; 5.13±1.54 kPa in F0-1, 7.42±2.53 kPa in F2, 12.18±3.00 kPa in F3, and 23.41±11.66 kPa in F4 for LSM and 0.16±0.08 in F0-1, 0.30±0.16 in F2, 0.61±0.23 in F3, and 0.75±0.20 in F4 for FT (all *p*<0.05 between adjacent fibrosis stages). The Spearman correlation coefficient of LSM and FT with histological fibrosis stages was 0.881 and 0.780, respectively (both *p*<0.001).

**Figure 3:**
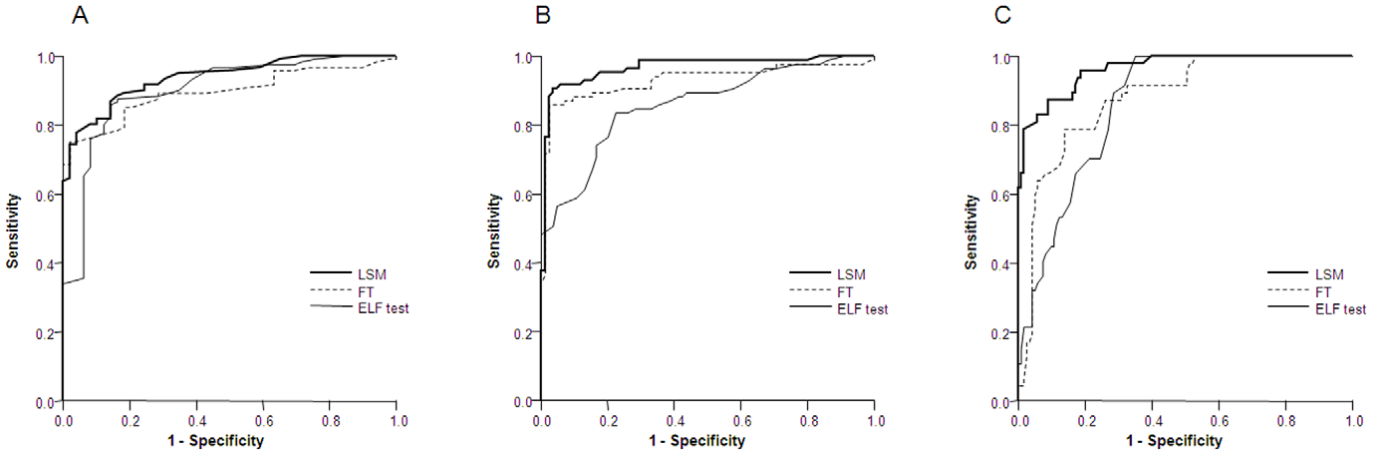
Receiver operating characteristic (ROC) curves for ELF test, LSM and FT in the diagnosis of significant fibrosis (≥F2, A), advanced fibrosis (≥F3, B), and cirrhosis (F = 4, C).

With regard to the diagnostic performances of ELF test for prediction of liver fibrosis, the AUROC was 0.901 (95% confidence interval [CI] 0.849–0.953) for significant fibrosis (F≥2) (**Figure 3A**), 0.860 (95% CI 0.805–0.915) for advanced fibrosis (F≥3) (**Figure 3B**), and 0.862 (0.809–0915) for cirrhosis (F = 4) (**Figure 3C**) (**Table 2**). ROC curves and AUROCs of LSM and FT are also shown (**Figure 3** and **Table 2**). The diagnostic performances of each component of ELF (HA, TIMP-1, and P3NP) and FT (α2-macroglobulin, haptoglobin, γ-GGT, bilirubin, and apolipoprotein A1) for prediction of significant fibrosis, advanced fibrosis, and cirrhosis are described in **Table S1**. Overall, the coefficient of variations (CV = standard deviation/mean) of ELF was 17.7%, while those of LSM and FT were 79.7% and 66.1%, respectively.

**Table 2:** Diagnostic performances of LSM, FT and ELF and their suggested optimal cutoff values.

Fibrosis stage	Method	AUROC (95% CI)	Cutoffs	Sensitivity (%)	Specificity (%)	PPV (%)	NPV (%)
	**ELF**	0.901 (0.849–0.953)	8.50	86.0	85.7	93.7	71.2
**F≥2**	**LSM**	0.937 (0.903–0.971)	8.0 kPa	77.7	95.9	97.9	63.5
	**FT**	0.896 (0.850–0.942)	0.31	75.2	97.9	98.9	61.5
	**ELF**	0.860 (0.805–0.915)	9.40	83.5	77.7	78.9	82.5
**F≥3**	**LSM** *	0.956 (0.929–0.983)	10.1 kPa	90.6	96.5	96.2	91.1
	**FT** *	0.921 (0.877–0.964)	0.51	85.8	97.7	97.3	87.4
	**ELF**	0.862 (0.809–0915)	10.10	70.2	78.9	55.9	87.4
**F = 4**	**LSM** *	0.963 (0.937–0.989)	14.0 kPa	87.2	91.1	78.8	94.9
	**FT**	0.881 (0.828–0.935)	0.67	78.7	78.8	68.5	91.4

Abbreviations: ELF, enhanced liver fibrosis; LSM, liver stiffness measurement; FT, FibroTest; AUROC, area under the receiver operating characteristics curve; CI, confidence interval; PPV, positive predictive value; NPV, negative predictive value.

* p<0.05 compared to AUROC of ELF using Hanley and McNeil test.

**Figure 4:**
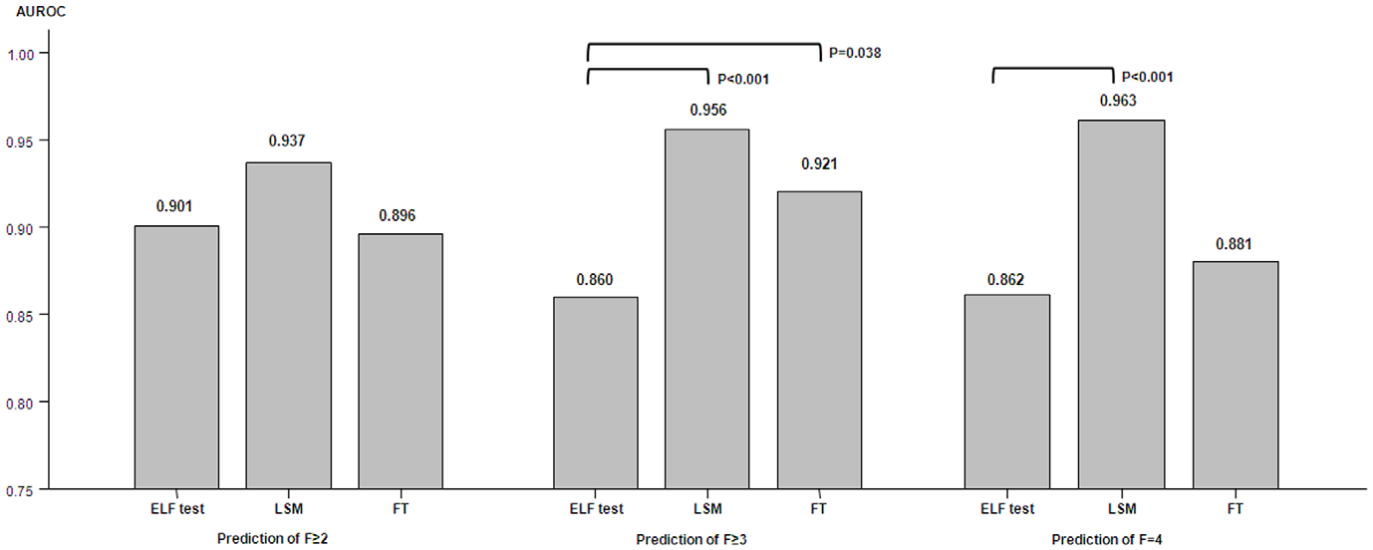
Detailed AUROCs of ELF test, LSM and FT in the diagnosis of significant fibrosis (≥F2), advanced fibrosis (≥F3), and cirrhosis (F = 4).

Among the three non-invasive methods, LSM consistently showed the highest AUROC for prediction of significant fibrosis, advanced fibrosis, and cirrhosis (**Figure 4** and **Table 2**). Although the accuracy of the three markers for prediction of significant fibrosis was statistically equivalent (all *p*>0.05), LSM showed significantly superior diagnostic efficacy over ELF test for predicting advanced fibrosis and cirrhosis (both *p*<0.001), and FT had better AUROCs than ELF test for predicting advanced fibrosis (F≥3) (p = 0.038) (**Figure 4** and **Table 2**).

### Determination of the optimal cutoffs for the ELF test

The most discriminant cutoff values for ELF test are indicated in **Table 2**. ELF cutoff values of 8.5, 9.40, and 10.10 generated a sensitivity of 86.0%, specificity of 85.7%, PPV of 93.7% and NPV of 71.2% for F≥2; sensitivity of 83.5%, specificity of 77.7%, PPV of 78.9%, and NPV of 82.5% for F≥3; sensitivity of 70.2%, specificity of 78.9%, PPV of 55.9%, and NPV of 87.4% for F = 4. The corresponding cutoff values of LSM and FT for each histological stage and their diagnostic indices are shown in detail in **Table 2**.

### Agreement between histological results and the ELF test, LSM, or FT

ELF test agreed with liver histology for fibrosis levels of F<2 *vs*. F≥2 in 146 (85.8%) patients, which was higher than LSM (n = 142, 83.5%) and FT (n = 138, 81.2%). However, the ELF predicted the fibrosis stage less accurately (confirmed histologically) for levels F<3 *vs*. F≥3 (n = 135, 79.4%) and F4 (n = 126, 74.1%) than did LSM (n = 158, 92.9% and n = 150, 88.2%, respectively) or FT (n = 155, 91.2% and n = 141, 82.9%, respectively) (**Table 3**).

**Table 3:** Distribution and agreement of fibrosis stages according to histology and ELF, LSM or FT (n = 170).

	Fibrosis stage estimated by ELF			
Fibrosis stage estimated by histology	F0-1	F2	F3	F4
	ELF<8.5	8.5≤ELF<9.4	9.4≤ELF<10.1	ELF≥10.10
**F1**	41	4	1	3
**F2**	7	12	9	8
**F3**	9	5	5	19
**F4**	0	0	14	33

	Fibrosis stage estimated by LSM			
Fibrosis stage estimated by histology	F0-1	F2	F3	F4
	LS<8.0 kPa	8.0≤LS<10.1 kPa	10.1≤LS<14.0 kPa	LS≥14.0 kPa
**F1**	45	3	1	0
**F2**	20	13	2	1
**F3**	4	3	18	13
**F4**	0	1	5	41

	Fibrosis stage estimated by FT			
Fibrosis stage estimated by histology	F0-1	F2	F3	F4
	FT<0.31	0.31≤FT<0.51	0.51≤FT<0.67	FT≥0.67
**F1**	47	2	0	0
**F2**	21	12	2	1
**F3**	5	1	14	18
**F4**	4	2	4	37

**Abbreviations**: ELF, Enhanced Liver Fibrosis; LSM, liver stiffness measurement; FT, FibroTest.

In addition, when using the suggested cutoffs of ELF test, LSM, and FT to diagnose each histological fibrosis stage (F1, F2, F3, and F4), 91 (53.5%), 117 (68.8%), and 110 (64.7%) patients (gray-colored area in **Table 3**) were correctly classified according to histological results, meaning that liver biopsy could have been replaced with these non-invasive methods (**Table 3**).

### Discordance between histological results and ELF test

Discordant results between fibrosis stages estimated by liver biopsy and ELF test were identified in 79 (46.5%) patients. On multivariate analysis, only the presence of histological cirrhosis was identified as a single significant factor, which was negatively associated with discordance between liver biopsy and ELF test (*p*<0.001; odds ratio 0.249, 95% CI 0.116–0.533), although, on univariate analysis, the mean ALT level differed significantly between patients with non-disconcordance and discordance (65 *vs*.76 IU/L, *p* = 0.035), along with the proportion of subjects with histological cirrhosis.

## Discussion

Most studies that proposed ELF test as a good non-invasive alternative to liver biopsy have focused primarily on populations with HCV infection or nonalcoholic fatty liver disease [18], [28] or those with mixed etiologies including viral hepatitis, primary biliary cirrhosis, *etc*. [19], [22], [29]. Since the diagnostic cutoffs of such non-invasive indices based on biochemical parameters can vary, even when the etiology is the same, possibly due to different distribution of fibrosis stages and different baseline biochemical profiles, a new study to generate standardized results in Asian patients with CHB is warranted. To the best of our knowledge, this is the first study to assess the diagnostic value of ELF test and to define new cutoff values for each fibrosis stage optimized for a homogenous Asian population with CHB.

Although the underlying mechanisms of fibrosis progression in chronic viral hepatitis are expected to be similar, differences according to etiology may affect the diagnostic accuracy of non-invasive tests [30], [31]. For example, patients with CHC often have steatosis, which may influence baseline biochemical parameters, and they also tend to have micronodular cirrhosis. In contrast, CHB patients frequently experience a wide range of fluctuations in necroinflammatory activity that can result in overestimation of liver fibrosis but also have macronodular cirrhosis, which can result in underestimation of liver fibrosis [2], [31]. These clinicopathological differences have been suggested to explain the different diagnostic performance and cutoff values of noninvasive markers such as LSM and FT among studies [9], [25], [32]. Hence, in the present study, we focused primarily on Asian patients with CHB and investigated the accuracy and applicability of ELF test in comparison with LSM and FT, which are the most popular indices used currently in clinical practice.

This study has several advantages. First, we prospectively recruited patients who underwent not only the baseline blood tests and LSM but also FT and ELF test on the same day as liver biopsy, and the diagnostic performance of ELF test was compared to those of LSM and FT, both of which are currently preferred in France over liver biopsy due to their excellent diagnostic values [10], and have also been recently validated in Asian populations with CHB [13], [24], [33], [34]. Furthermore, a relatively large number of subjects from a single center were consecutively enrolled in this study, and the distribution of our population was homogeneous and representative of patients with CHB seen in clinical practice. Therefore, the optimal cutoff values of ELF test derived from our study are expected to ultimately be used as reference values for future studies to elaborate its role in Asian patients with CHB. Last, we considered only biopsy specimens of 20 mm or larger. Given that intra- and inter-observer variability may exist in histological assessment of fibrosis staging, obtaining specimens of adequate size is of utmost importance to ensure the greatest possible degree of uniformity [4].

In the present study, the diagnostic performance of ELF test was comparable to that of the LSM or FT for predicting significant fibrosis (F≥2); AUROC 0.901 *vs*. 0.937 or 0.896. This is consistent with several other reports [12], [35], suggesting that non-invasive tests have similar accuracy for detection of significant fibrosis each other. However, LSM consistently had significantly better performance than ELF test in predicting both advanced fibrosis (F≥3) (AUROC 0.956 *vs*. 0.860) and cirrhosis (F = 4) (AUROC 0.963 *vs*. 0.862). A recent meta-analysis demonstrated a similar finding, that LSM may be much more accurate for cirrhosis than for less severe fibrosis stages [16]. Regarding comparison between ELF test and FT, the diagnostic value of ELF test was significantly lower than that of FT for diagnosis of advanced fibrosis (F≥3) (0.860 *vs*. 0.921); however, the performance of the ELF test and FT for diagnosing significant fibrosis (F≥2) (0.901 *vs*. 0.896) and cirrhosis (F4) (0.862 *vs*. 0.881) was actually equivalent (all *p*>0.05). In contrast, Friedrich-Rust *et al*. [19] proposed that ELF test (AUROC 0.91) can diagnose cirrhosis with comparable diagnostic accuracy to LSM (AUROC 0.94) and FT (AUROC 0.92). However, this study was limited in that only a small portion of the study cohort underwent both LSM and ELF. On the other hand, another recent study [21] insisted that LSM showed greater accuracy than ELF for detecting significant fibrosis and cirrhosis. Taken together, given these controversial results, the performance of ELF test as a non-invasive alternative compared to LSM or FT should be further validated.

Using the Youden method [27], we suggested ELF cutoff values of 8.5, 9.4, and 10.1 for F≥2, F≥3, and F = 4, respectively. However, Parkes *et al*. [18] reported 10.2 with a maximum sum of sensitivity (70%) and specificity (85%) for diagnosis of F≥3, while Guecho *et al*. [36] suggested 9.00, 9.33, and 9.35 for F≥2, F≥3, and F = 4, respectively. The suggested thresholds and intervals between adjacent stages may vary among studies, and they may be influenced by etiologies, sample size, and the baseline characteristics of populations. Thus, further external investigations with larger sample sizes and a balanced fibrosis stage distribution are needed to validate our data in Asian subjects with CHB.

Regarding discordant results between the histological examination and ELF test, the presence of histological cirrhosis ultimately proved to be a single significant factor with a negative association with the discordant results. Although the lower trend of ALT level in those with non-discordance compared to those with discordance seen on univariate analysis (mean value 65 vs. 76 IU/L, respectively; *p* = 0.035) did not reduce the discordance rate independently on multivariate analysis, it might be inferred that this negative correlation between the presence of histological cirrhosis and discordances can be in part associated with the different level of ALT in patients with and without histological cirrhosis (mean value 46 vs. 85 IU/L, respectively; *p* = 0.039), as observed in several related studies [13], [37], [38]. Thus, further studies are required to elucidate the possible confounding variables of ELF test.

This study did have some limitations. First, as this was a cross-sectional study, it is not clear whether repeated determination of ELF score may be useful for tracking the progression of fibrosis and related clinical outcomes, such as occurrence of hepatic decompensation and HCC in individual patients. Therefore, the diagnostic value for prediction of the subsequent development of cirrhosis and its various complications with sequential ELF tests during long-term follow-up must be examined further in a longitudinal study. Second, our population included a small portion of patients with F0 fibrosis. This could have resulted in a selection bias and eventually a spectrum bias, since the diagnostic performance of a given noninvasive test may depend on the disease prevalence. Since our institute is a tertiary referral hospital and one of the largest medical centers in South Korea, cases with advanced disease are likely to be referred for close observation. When we further analyzed the performance of ELF to distinguish F≥1 from F0, the AUROC was also high (0.904, CI 0.841–0.967) with an optimal cutoff value of 8.15. However, this result should be applied in real clinical practice cautiously, considering the small proportion (only 5.9%) of patients with F0 among entire population. Taken together, further studies for external validation based upon a community-based cohort should be performed to provide more generalized results for patients with CHB. Third, if we had enrolled more subjects, the diagnostic accuracy of ELF would have been much better. Indeed, when we added the clinical data of 36 patients with CHB who were further recruited for additional 4 months, concordance rate between fibrosis stages based on histology and ELF had become enhanced up to 61.7% (vs. 53.5%; **Table S2**). Thus, a large-scale study should be followed to provide the solid evidences.

In summary, in a prospective study, we first assessed ELF test in Asian patients with CHB, demonstrating its considerable diagnostic value for prediction of histological fibrosis stage, and the optimal suggested cutoff values are expected to be useful in future studies in those populations. However, LSM consistently provided better diagnostic values in the higher fibrosis stages. We hope that other researchers will evaluate the reproducibility of ELF test and its potential role as a method of classifying liver fibrosis in independent populations.

## Supporting Information

Table S1 Diagnostic performance (AUROC) of each component for FT and ELF.
(DOCX)

Table S2 Distribution and agreement of fibrosis stages according to histology and ELF (n = 206).
(DOCX)
