# Supplementary material for: Prospective Validation of ELF Test in Comparison with Fibroscan and FibroTest to Predict Liver Fibrosis in Asian Subjects with Chronic Hepatitis B
Source: PLoS One. 2012 Jul 27;7(7):e41964. doi: 10.1371/journal.pone.0041964 (PMC3407050; doi:10.1371/journal.pone.0041964)
Supplement: Table S1 — Diagnostic performance (AUROC) of each component for FT and ELF. (DOCX) [file pone.0041964.s001.docx]

| **Supplementary table 1. Diagnostic performance (AUROC) of each component for FT and ELF** | | | |
| --- | --- | --- | --- |
|  | F≥2 | F≥3 | F4 |
| α2-macroglobulin | 0.783 (0.667-0.899) | 0.775 (0.685 - 0.864) | 0.665 (0.600 - 0.762) |
| haptoglobin | 0.754 (0.641 - 0.867) | 0.699 (0.607 - 0.792) | 0.697 (0.601 - 0.794) |
| γ-GGT | 0.765 (0.650 - 0.880) | 0.745 (0.652 - 0.837) | 0.734 (0.644 - 0.823) |
| bilirubin | 0.748 (0.639 - 0.857) | 0.732 (0.640 - 0.825) | 0.733 (0.637 - 0.828) |
| apolipoprotein A1 | 0.705 (0.609 - 0.819) | 0.677 (0.607 - 0.792) | 0.623 (0.600 - 0.726) |
|  |  |  |  |
| HA | 0.902 (0.820-0.952) | 0.865 (0.694-0.834) | 0.896 (0.851-0.942) |
| TIMP1 | 0.607 (0.513-0.701) | 0.673 (0.593-0.754) | 0.657 (0.571-0.742) |
| P3NP | 0.799 (0.729-0.870) | 0.764 (0.694-0.834) | 0.719 (0.639-0.799) |
