# Supplementary material for: Prospective Validation of ELF Test in Comparison with Fibroscan and FibroTest to Predict Liver Fibrosis in Asian Subjects with Chronic Hepatitis B
Source: PLoS One. 2012 Jul 27;7(7):e41964. doi: 10.1371/journal.pone.0041964 (PMC3407050; doi:10.1371/journal.pone.0041964)
Supplement: Table S2 — Distribution and agreement of fibrosis stages according to histology and ELF (n = 206). (DOCX) [file pone.0041964.s002.docx]

| **Supplementary table 2. Distribution and agreement of fibrosis stages according to histology and ELF (n=206)** | | | | | |
| --- | --- | --- | --- | --- | --- |
|  | **Fibrosis stage estimated by ELF** | | | |  |
| **Fibrosis stage estimated by histology** | **F0-1** | **F2** | **F3** | **F4** |  |
|  | **ELF < 8.5** | **8.5 ≤ ELF < 9.4** | **9.4 ≤ ELF < 10.1** | **ELF ≥ 10.10** |  |
| **F1** | 62 | 4 | 1 | 3 |  |
| **F2** | 7 | 15 | 9 | 8 |  |
| **F3** | 9 | 5 | 6 | 19 |  |
| **F4** | 0 | 0 | 14 | 44 |  |
